# Impact of the number of lymph node dissections and a novel risk stratification on the prognosis in elderly locally advanced esophageal adenocarcinoma

**DOI:** 10.7150/jca.96574

**Published:** 2024-06-03

**Authors:** Yahua Wu, Weiwei Gu, Bin Du, Chengliu Lv, Na Yao, Yingjiao Zhu, Jingxiang Ouyang, Jinhuo Lai

**Affiliations:** 1 Department of Medical Oncology, Fujian Medical University Union Hospital, Fuzhou, 350000, Fujian, China.; 2 Department of Medical Oncology, People's Hospital Affiliated to Shandong First Medical University, Jinan, 271100, Shandong, China.; 3 Department of Medical Oncology, Zhenghe County Hospital, Nanping, 353699, Fujian, China.

**Keywords:** Esophageal adenocarcinoma, Neoadjuvant chemoradiotherapy, Lymph node dissection, Risk score, SEER

## Abstract

**Background:** Elderly patients with locally advanced esophageal adenocarcinoma (EAC) have a poor prognosis. The purpose of this study was to identify prognostic factors and construct a risk stratification for assessing the prognosis of elderly (≥ 70 years old) EAC patients who receiving neoadjuvant chemoradiotherapy (NCRT) and esophagectomy.

**Methods:** A total of 688 patients with non-metastatic locally advanced EAC who underwent NCRT and esophagectomy were selected from the Surveillance Epidemiology and End Results (SEER) database. Multivariable Cox analysis was used to identify prognostic factors of overall survival (OS). Restricted Cubic Splines (RCS) was used to examine the linear relationship between the number of lymph node dissection (LND) and OS.

**Result:** RCS showed a linear relationship between LND and OS (P = 0.690). As the number of LND increased, the risk of death decreased. Multivariable analysis demonstrated that LND > 23, grade III/IV, and regional node positive were independent prognostic factors. Subgroup analysis indicated that enlarged lymph node dissection (LND > 23) did not improve OS in patients with grade I/II or T1-2 stage, whereas enlarged lymph node dissection significantly improved OS in patients with grade III/IV or T3-4 stage. Furthermore, we constructed a novel risk score based on LND, grade, and regional node status, which stratified patients into low-, medium-, and high-risk groups. Patients in the high-risk group (risk score = 3) had a worse prognosis.

**Conclusions:** Enlarged lymph node dissection (LND > 23) improved OS in patients with grade III/IV or T3-4 stage. Moreover, a novel risk score was constructed, which facilitated risk stratification and postoperative surveillance in elderly EAC patients.

## 1. Introduction

Esophageal cancer remains one of the malignant tumors with high morbidity and mortality in the world.^1^ China accounts for approximately 50% of global esophageal cancer cases.^2^ Esophageal adenocarcinoma (EAC) is a prevalent histopathological subtype with a growing annual incidence rate. A large proportion of patients with EAC are diagnosed at advanced stages of the disease.^3^

The CROSS clinical study was a landmark event, marking neoadjuvant chemoradiotherapy (NCRT) followed by esophagectomy as the standard treatment modality for locally advanced resectable EAC. However, similar to most clinical studies, the median age of the population was approximately 60 years old in the CROSS study.^4^, ^5^ Subsequently, a growing number of retrospective studies have demonstrated that older patients with esophageal cancer can also benefit from this trimodality therapy.^6^, ^7^ Therefore, for elderly patients with locally advanced EAC, neoadjuvant chemoradiotherapy followed by esophagectomy remains the recommended first-line treatment strategy. However, the debate over the extent of lymph node dissection has been ongoing. Some studies suggested that more lymph nodes cleared after neoadjuvant chemoradiotherapy was associated with better survival outcomes.^8^ There were also studies that had come to the opposite conclusion that the number of lymph nodes removed did not improve patients' survival.^9^ Nevertheless, there is a lack of studies reporting on the correlation between the extent of lymph node dissection and survival outcomes in elderly patients with EAC who received NCRT.

Therefore, the aim of our study was to investigate prognostic factors of elderly EAC patients (≥ 70 years old), to explore the effect of the number of lymph node dissection on overall survival, and to construct a new risk stratification in order to guide clinical decision-making for locally advanced elderly EAC patients who treated with NCRT and surgery.

## 2. Methods

### 2.1 Patients selection

**Figure 1:**
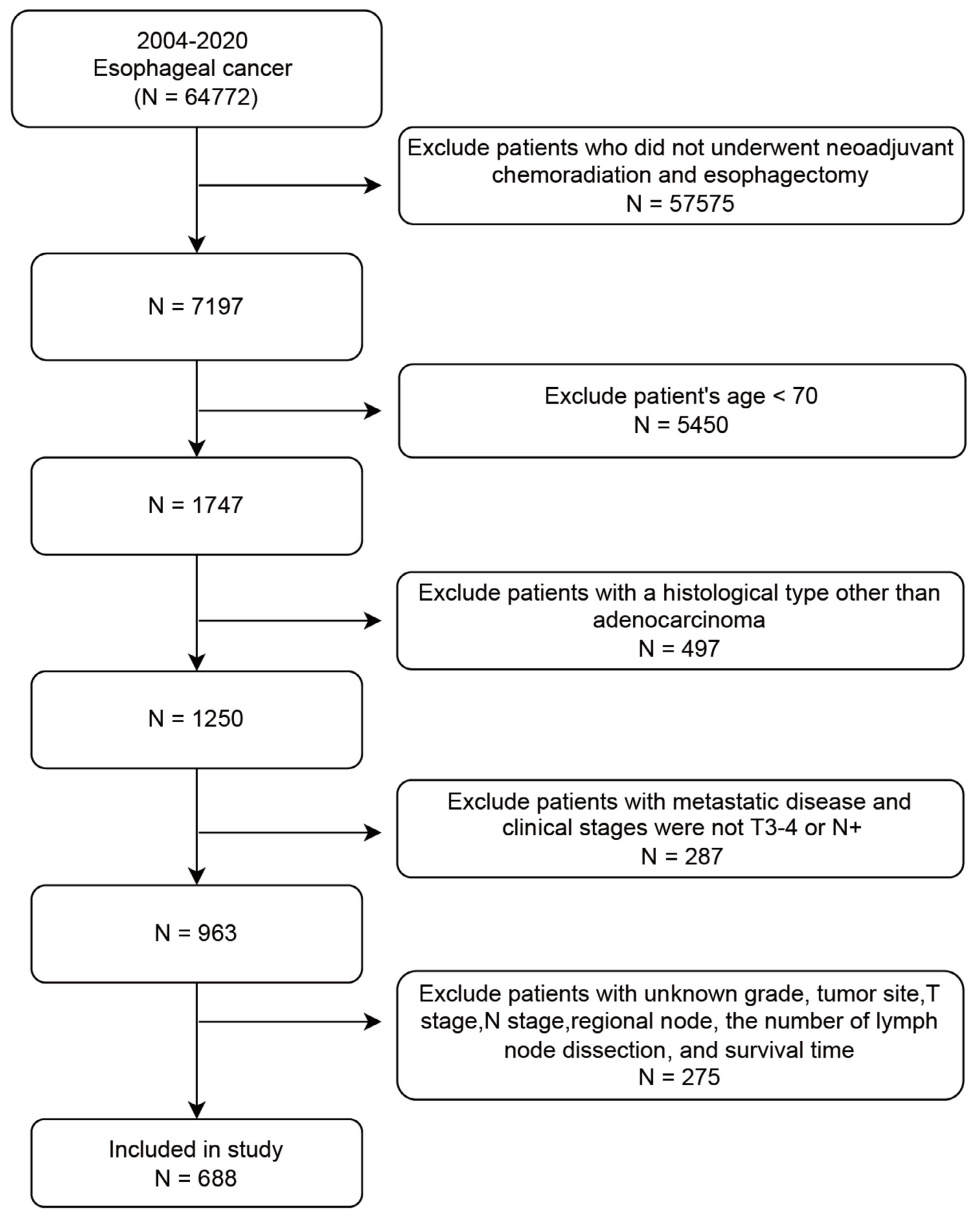
The patient screening workflow diagram.

The SEER database collected cancer incidence data from population-based cancer registries covering approximately 48.0 percent of the United States (http://seer.cancer.gov/). We enrolled 688 elderly patients (≥ 70 years) with non-metastatic locally advanced EAC who underwent NCRT followed by surgery between 2004 and 2020 from the SEER database. The covariates included age, gender, tumor site, grade, T stage, N stage, regional node status, and lymph nodes dissection (LND). The patient screening workflow diagram is shown in Figure 1. The inclusion criteria: (1) year of diagnosis from 2004 to 2020; (2) diagnosed as esophageal carcinoma; (3) clinical staging at locally advanced stage (T3/4 or N+ and M0). The exclusion criteria: (1) patients who did not undergo neoadjuvant radiotherapy (beam radiation), chemotehrapy, and esophagectomy; (2) age < 70 years; (3) patients with a histological type other than adenocarcinoma. The specific ICD-O3 code for esophageal adenocarcinoma was 8140; (4) patients with metastatic disease; (5) patients with unknown data such as gender, tumor site, T stage, N stage, regional node status, the number of LND, and survival time.

### 2.2 Statistical analysis

All statistical calculations were analyzed using SPSS (Version 25.0) and R software (Version 4.0.2). The cutoff value for the number of LND was calculated using X-tile software (Version 3.6.1), which was a valuable tool to generate the optimal cut-point with minimum p values.^10^ The relationship between the number of LND and OS was evaluated using restricted cubic splines (RCS), which could reveal the true nature and complexity of the relationships between continuous variables.^11^ Survival curves were estimated using the Kaplan-Meier method and compared by the log-rank test. The time-dependent area under the receiver operating characteristic (ROC) curve (AUC) was used to evaluate the risk score. All factors with a p-value < 0.05 in univariate Cox regression analysis were entered into multivariate Cox regression analysis to determine independent prognostic factors. All statistical analyses were two-sided, and significance was defined as P < 0.05.

## 3. Results

### 3.1 Patients characteristics

A total of 688 elderly EAC patients who underwent NCRT followed by surgery met inclusion criteria. As shown in Table 1, there were 267 (38.3%) patients who were older than 75 years. The majority of patients were male (88.1%), lower esophageal cancer (95.1%), T3-4 stage (84.3%), and N+ stage (76.0%). The median LND number is 15. In addition, regional lymph node was positive in 41.4% of patients and negative in 58.6% of patients.

### 3.2 The relationship between LND and OS

**Figure 2:**
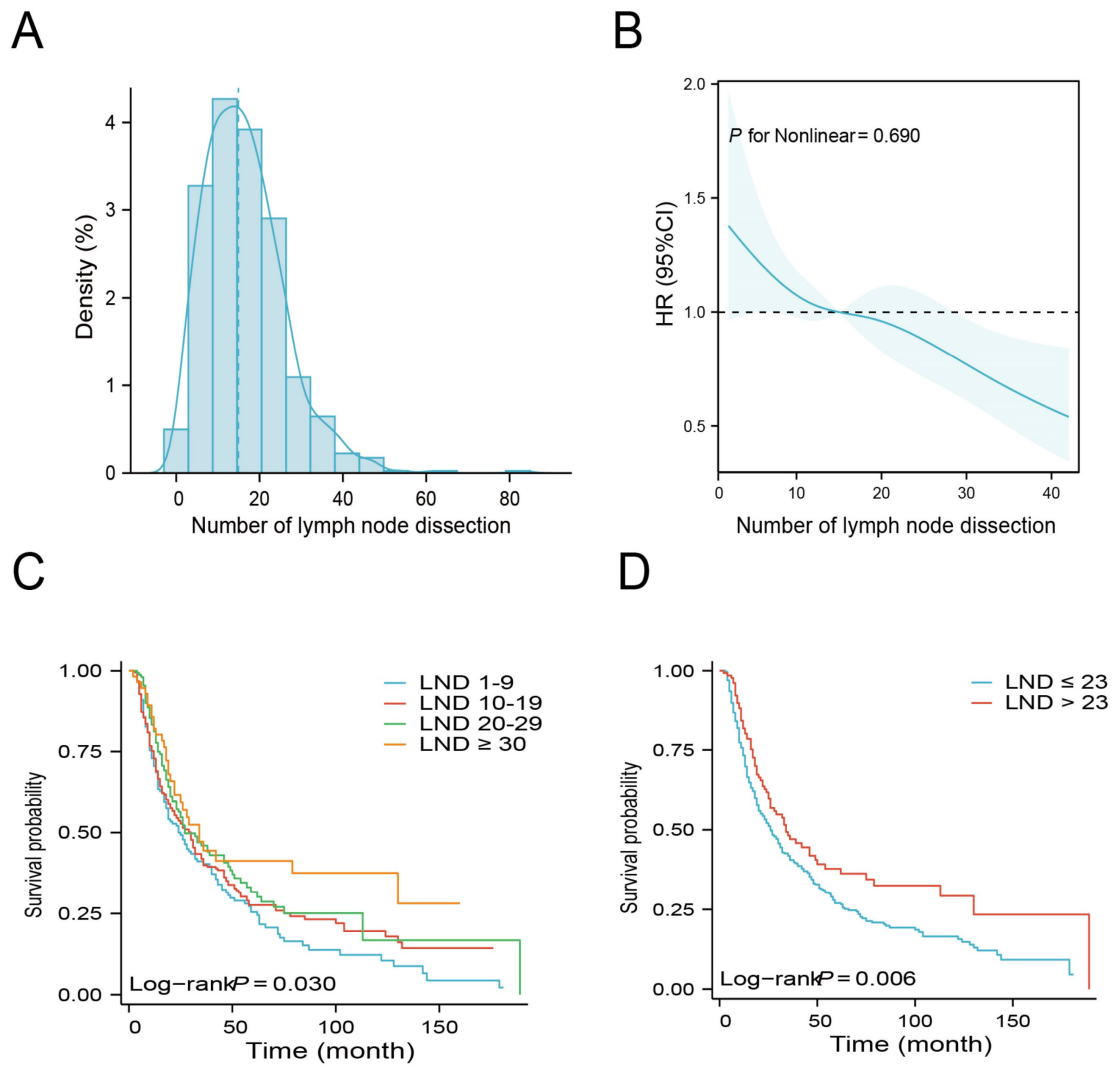
The relationship between the number of lymph node dissection (LND) and overall survival (OS). Distribution of the number of LND (A). Restricted Cubic Spline analysis was used to classify the association between the number of LND and mortality risk (B). Kaplan-Meier curves for OS according to the number of LND (1-9 vs. 10-19 vs. 20-29 vs. ≥ 30) (C). Kaplan-Meier curves for OS according to the number of LND (≤ 23 vs. >23) (D).

Figure 2A illustrates the density plot that presents the distribution of the number of LND across the entire cohort. An analysis using Restricted Cubic Spline (RCS) was conducted, adjusting for age, gender, tumor site, grade, T stage, N stage, and regional lymph node status to assess the relationship between LND and mortality risk. Figure 2B depicts a linear correlation between LND and OS, with a statistically insignificant nonlinear p-value of 0.690, indicating a reduced risk of mortality with an increase in the number of lymph nodes removed. Based on the number of LND, patients were categorized into 1-9, 10-19, 20-29, and ≥ 30. Patients with LND ≥ 30 had the best survival, with a median OS of 34 months and a 5-year OS rate of 41.3%. Conversely, patients with LND 1-9 had the worst survival with a median OS of 24 months and a 5-year overall survival rate of 25.5% (Table 2). The Kaplan-Meier survival curves shows that patients with LND ≥30 exhibit a superior OS compared to other groups. (Figure 2C, P = 0.03).

**Figure 3:**
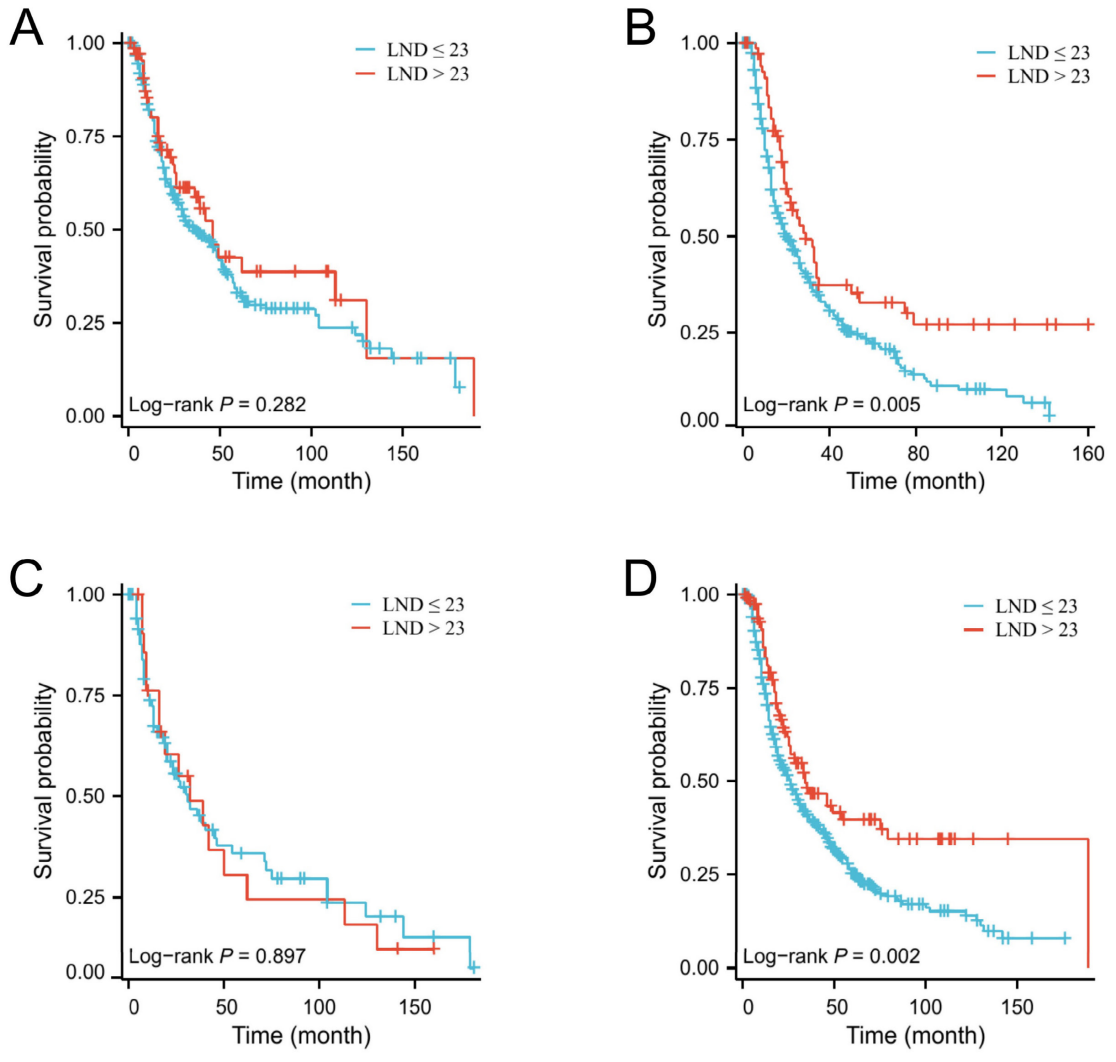
The effect of the number of lymph node dissection on overall survival in elderly EAC patients with grade I/II (A), grade III/IV (B), T1-2 stage (C) and T3-4 stage (D).

Next, the X-tile software was used to determine the optimal number of LND. The results indicated that the optimal cutoff value of the number of LNDs was 23. Then, patients were divided into ≤ 23 and > 23 groups. There were no significant differences in baseline clinical characteristics between these two groups (Table 3). Kaplan-Meier survival curves show that patients with LND > 23 have a better median OS than those with LND ≤ 23 (Figure 2D, P = 0.006). Subgroup analysis indicated that enlarged lymph node dissection (LND > 23) did not significantly improve OS in patients with grade I/II or T1-2 stage, whereas enlarged lymph node dissection significantly improved OS in patients with grade III/IV or T3-4 stage (Figure 3).

### 3.3 Factors associated with OS

In univariate Cox analysis, the prognostic factors for OS included male (HR: 1.414, 95% CI: 1.031 - 1.938, P = 0.031), grade III/IV (HR: 1.498, 95% CI: 1.235 - 1.817, P < 0.001), N1-3 (HR: 1.438, 95% CI: 1.139 - 1.815, P = 0.002), LND > 23 (HR: 0.702, 95% CI: 0.545 - 0.905, P = 0.006), and regional lymph node positive (HR: 1.913, 95% CI: 0.1.581 - 2.314, P = < 0.001). Multivariate Cox analysis confirmed that grade III/IV (HR: 1.516, 95% CI: 1.248 - 1.840, P < 0.001), LND > 23 (HR: 0.650, 95% CI: 0.504 - 0.839, P < 0.001), and regional lymph node positive (HR: 1.916, 95%CI: 1.536 - 2.391, P < 0.001) were independent prognosticators (Table 4).

### 3.4 Risk stratification for elderly EAC

**Figure 4:**
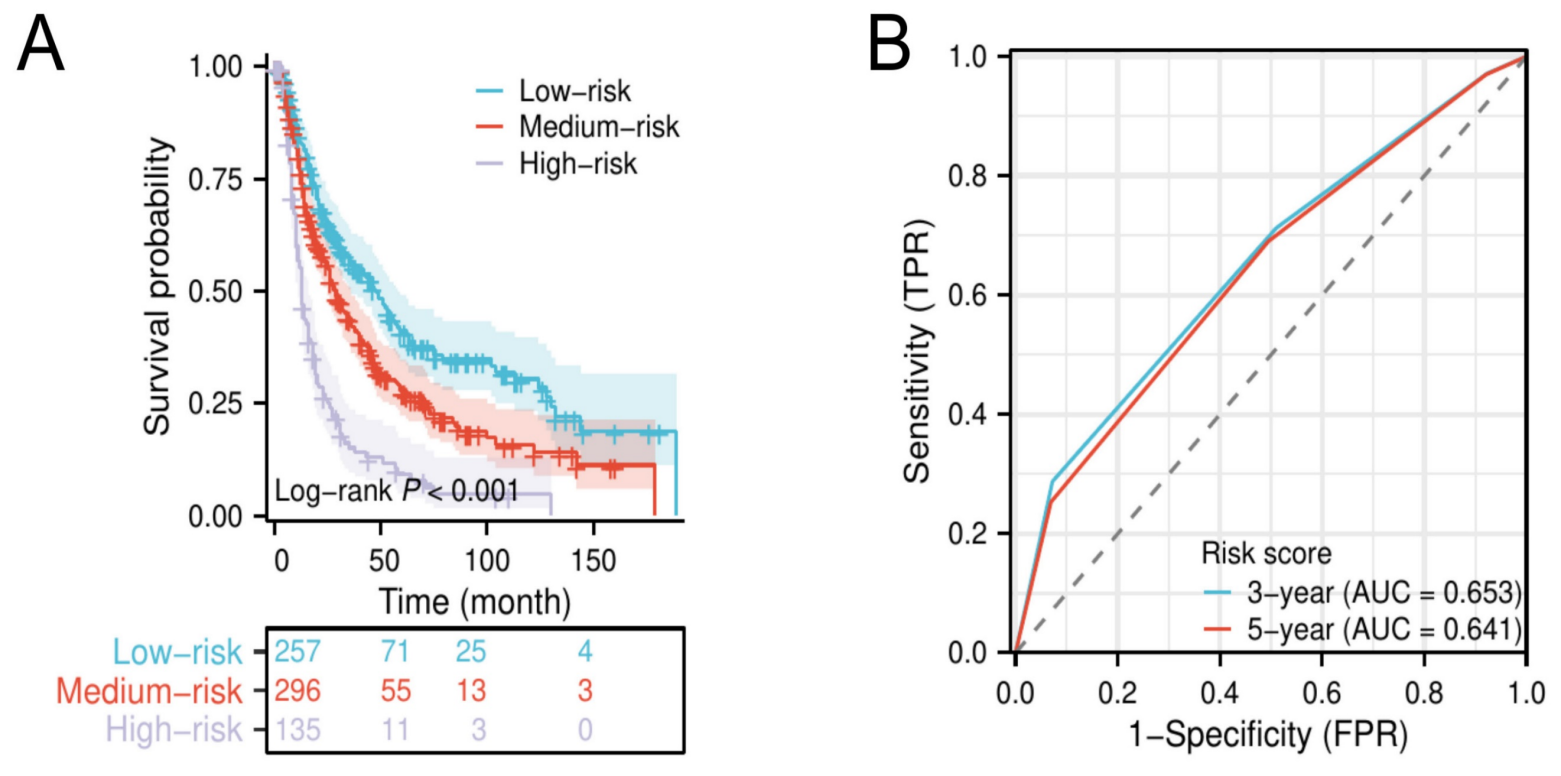
Kaplan-Meier survival curves for elderly EAC patients in low-, medium-, and high-risk groups (A). Time-dependent ROC of the risk score for predicting 3- and 5-year overall survival (B).

The results of the multivariate Cox analysis suggested that grade III/IV, LND ≤ 23, and regional lymph node positive were independent unfavorable prognostic factors for elderly patients with locally advanced EAC. Then, we defined grade III/IV, LND ≤ 23, and regional lymph node positive as a score of 1, respectively, and established a novel risk score to stratify patients into low- (0-1 score), medium- (2 scores), and high-risk groups (3 scores). Kaplan-Meier survival analysis shows that patients in the high-risk group had a significantly lower median OS compared to patients in the low- and medium-risk groups (Figure 4A). The time-dependent ROC curves show that the AUC values of the risk score for 3‐year and 5‐year were 0.653 and 0.641, respectively (Figure 4B).

## 4. Discussion

Neoadjuvant chemoradiotherapy followed by esophagectomy is considered the standard treatment for locally advanced resectable EAC.^5^ However, few studies have reported factors associated with prognosis in elderly EAC patients who received neoadjuvant chemoradiotherapy and surgery. We included 688 elderly EAC patients (≥70 years old) from the SEER database. The multivariate analysis revealed that grade, the number of lymph node dissection (LND), and regional lymph node status were independent prognostic factors for elderly EAC patients who treated with neoadjuvant chemoradiotherapy and esophagectomy.

The number of lymph node dissections is an important prognostic factor for resected EAC patients.^12^, ^13^ However, the number of LND in locally advanced EAC patients who undergo neoadjuvant therapy remains controversial. Most studies concluded that extending the number of LND improved the patient's prognosis. Hanna *et al.* demonstrated that patients with a higher number of lymph nodes removed had a longer survival time.^8^ Similarly, a large cohort study proved that enlarged LND was associated with better survival time, suggesting the need for expanded lymph node dissection during esophagectomy.^14^ When the number of lymph nodes removed is insufficient, potentially metastatic lymph nodes may not be detected resulting in recurrence of the tumor. Micrometastases have been reported in up to 50% of patients with histologically negative lymph nodes.^15^ Expanded lymph node dissection may eliminate occult lymph nodes, reduce local recurrence rates, and then improve survival rates.^16^-^18^ However, there are some studies that have come up with opposite results. They concluded that extended lymph node dissection did not improve patients' survival after neoadjuvant therapy. A study by Talsma *et al.* including 161 patients who underwent surgery alone and 159 patients who treated with NCRT plus surgery showed that the number of lymph nodes resected had an impact on the prognosis of patients who underwent surgery alone, but not those who underwent NCRT.^19^ Another study also indicted that the number of lymph nodes removed did not improve patients' survival after NCRT.^9^

Elderly patients are a special group that is excluded from clinical trials due to their poor physical condition and the presence of other medical diseases.^4^, ^20^ Currently, there is limited research on the impact of the number of lymph nodes removed on the prognosis of elderly patients with esophageal cancer. A retrospective study by Zhang *et al.* revealed that extensive lymphadenectomy significantly improved survival for non-elderly patients, but it did not affect the survival of elderly patients (≥75 years).^21^ However, this study excluded patients with esophageal cancer receiving neoadjuvant therapy. There is a lack of studies focusing on the effect of the number of lymph node dissections on elderly EAC patients after neoadjuvant therapy. Our findings demonstrated that a number of lymph node dissections greater than 23 indicated significantly a better OS in elderly EAC patients who underwent NCRT. Multivariate analysis further confirmed that the number of lymph node dissections was an independent prognostic factor for locally advanced elderly EAC patients after neoadjuvant therapy.

In addition, we further analyzed the relationship between the number of LND and OS using restricted cubic spline, which showed a linear correlation between the number of LND and the risk of death, with a decrease in the risk of death as the number of LND increased. When the number of lymph nodes cleared was less than 23, the risk of death showed a dramatic increase, suggesting that adequate lymph node dissection is necessary in elderly patients after receiving neoadjuvant therapy. Many previous studies have demonstrated that extended lymph node dissection improved survival in patients with esophageal cancer after neoadjuvant chemoradiotherapy, but the median age of patients included in these studies was approximately 60 years.^8^, ^14^ Our study yielded similar results, suggesting that expanded lymph node dissection could improve older (≥ 70 years) EAC patients' survival after neoadjuvant therapy. Therefore, it has been speculated that (besides potentially removing micro metastatic disease), an enlarged lymph node dissection may be a surrogate for better surgery therefore better long-term outcomes after a large complex surgical procedure.

However, in the era of immunotherapy, there is a renewed debate about the necessity of expanded lymph node dissection at the time of esophagectomy. The CheckMate 577 study has confirmed that adjuvant immunotherapy is the standard of care for patients with EAC who undergo surgery after neoadjuvant chemoradiation therapy and have residual adenocarcinoma in their pathologic specimen.^22^ However, similar to most clinical studies, the median age of patients included in this study was around 60 years, and it is still questionable whether adjuvant immunotherapy benefits older patients. Secondly, in general, elderly patients have low immune function that results in poorer efficacy of immunotherapy. A study found that the number of immune cells changed with age, which manifested in the increase of virtual memory T-cells and the loss of primary T-cells,^23^ that explained the poor efficacy of immunotherapy in older patients. Thus, the benefit of adjuvant immunotherapy in elderly EAC patients is limited. Instead, extended lymph node dissection may be more beneficial for local control of the tumor and prognosis. Additionally, extended lymph node dissection can help in accurate staging,^24^ which also provides a basis for adjuvant immunotherapy.

Moreover, our study also found that the grade was not only an important prognostic indicator for elderly EAC patients, but also as an essential indicator of the number of lymph nodes cleared during esophagectomy. We performed subgroup analyses and found that expanded lymph node dissections (LND > 23) did not improve OS in grade I/II patients, while it significantly improved OS in grade III/IV patients. Furthermore, for patients with stage T1-2, extended lymph node dissections also did not improve OS, whereas for patients with T3-4, extended lymph node dissections could significantly prolong OS. Therefore, it is reasonable to suggest that expanding the number of lymph node dissections after neoadjuvant therapy is still warranted for elderly EAC patients with poorly differentiated, T3-4 stage.

Besides, regional lymph node status also significantly influenced the prognosis of locally advanced elderly EAC patients. Based on LND, grade, and regional lymph node status, we constructed a novel risk score to stratify patients into low- (0-1 score), medium- (2 scores), and high-risk groups (3 scores). When patients were in the high-risk group (risk score = 3 points), their overall survival time was significantly decreased, and active postoperative monitoring was necessary since early salvage measures might be able to improve patients' survival.

Finally, our study had a relatively large sample size of elderly EAC patients, and we only included adenocarcinoma to minimize the confounding effect of other histology on our results. However, our study also had several limitations. First of all, this study was a retrospective study with an underlying weakness. Secondly, the SEER database did not provide specific description on radiation methods, chemotherapy regimen and esophagectomy skills. Thirdly, the data for the current study was obtained from the SEER database and there was a lack of validation cohort to confirm our conclusions. Furthermore, prospective studies were needed to validate our results.

## 5. Conclusion

The number of LND, grade, and regional lymph node status significantly affected survival in elderly patients with locally advanced EAC after neoadjuvant therapy. Expanded lymph node dissection (LND > 23) did not improve OS in patients with grade I/II or T1-2 stage, but significantly prolonged OS in patients with grade III/IV or T3-4 stage. Furthermore, a novel risk score was constructed based on the number of LND, grade, and regional lymph node status. Patients in the high-risk group (risk score = 3) had a significantly lower prognosis and might require adjuvant therapy as well as active surveillance.

## Figures and Tables

**Table 1:** Baseline characteristics of elderly patients with locally advanced esophageal adenocarcinoma

Characteristics	N = 688
Age, n (%)	
70-74	421 (61.2%)
≥75	267 (38.8%)
Gender, n (%)	
Male	606 (88.1%)
Female	82 (11.9%)
Tumor site, n (%)	
Upper/middle	34 (4.9%)
Lower	654 (95.1%)
Grade, n (%)	
I/II	311 (45.2%)
III/IV	377 (54.8%)
T stage, n (%)	
T1-2	108 (15.7%)
T3-4	580 (84.3%)
N stage, n (%)	
N0	165 (24.0%)
N1-3	523 (76.0%)
Lymph nodes dissection, median (IQR)	15 (9, 22)
Regional lymph node, n (%)	
Negative	403 (58.6%)
Positive	285 (41.4%)

**Table 2:** Survival analysis of different numbers of lymph node dissection

Variable	Median (month)	95% CI (month)		5-year overall survival rate (%)	P value
		Lower	Upper		
Number of lymph node dissection					0.030
1-9	24	19	33	25.5	
10-19	30	22	35	27.7	
20-29	27	23	48	31.7	
≥ 30	34	22	-	41.3	

**Table 3:** Baseline characteristics of patients between lymph node dissection ≤ 23 and > 23.

Characteristics	Lymph node dissection ≤ 23 (n = 550)	Lymph node dissection > 23 (n = 138)	P value
Age, n (%)			0.618
70-74	334 (60.7%)	87 (63%)	
≥75	216 (39.3%)	51 (37%)	
Gender, n (%)			0.871
Male	485 (88.2%)	121 (87.7%)	
Female	65 (11.8%)	17 (12.3%)	
Tumor site, n (%)			0.215
Upper/middle	30 (5.5%)	4 (2.9%)	
Lower	520 (94.5%)	134 (97.1%)	
Grade, n (%)			0.282
I/II	243 (44.2%)	68 (49.3%)	
III/IV	307 (55.8%)	70 (50.7%)	
T, n (%)			0.930
T1-2	86 (15.6%)	22 (15.9%)	
T3-4	464 (84.4%)	116 (84.1%)	
N, n (%)			0.807
N0	133 (24.2%)	32 (23.2%)	
N1-3	417 (75.8%)	106 (76.8%)	
Regional lymph node, n (%)			0.350
Negative	327 (59.5%)	76 (55.1%)	
Positive	223 (40.5%)	62 (44.9%)	

**Table 4:** Univariate and multivariate analysis for elderly EAC patients

Characteristics	Univariate analysis			Multivariate analysis	
	Hazard ratio (95% CI)	P value		Hazard ratio (95% CI)	P value
Age					
70-74	Reference				
≥75	1.150 (0.948 - 1.396)	0.156			
Gender					
Female	Reference			Reference	
Male	1.414 (1.031 - 1.938)	0.031		1.280 (0.933 - 1.758)	0.126
Tumor site					
Upper/middle	Reference				
Lower	1.047 (0.703 - 1.559)	0.820			
Grade					
I/II	Reference			Reference	
III/IV	1.498 (1.235 - 1.817)	< 0.001		1.516 (1.248 - 1.840)	< 0.001
T stage					
T1-2	Reference				
T3-4	1.094 (0.844 - 1.419)	0.498			
N stage					
N0	Reference			Reference	
N1-3	1.438 (1.139 - 1.815)	0.002		1.016 (0.776 - 1.330)	0.909
Lymph node dissection					
≤ 23	Reference			Reference	
>23	0.702 (0.545 - 0.905)	0.006		0.650 (0.504 - 0.839)	< 0.001
Regional lymph node					
Negative	Reference			Reference	
Positive	1.913 (1.581 - 2.314)	< 0.001		1.916 (1.536 - 2.391)	< 0.001

## References

[1] Sung H, Ferlay J, Siegel RL, Laversanne M, Soerjomataram I, Jemal A (2021). Global Cancer Statistics 2020: GLOBOCAN Estimates of Incidence and Mortality Worldwide for 36 Cancers in 185 Countries. CA Cancer J Clin.

[2] Zhu H, Ma X, Ye T, Wang H, Wang Z, Liu Q (2023). Esophageal cancer in China: Practice and research in the new era. Int J Cancer.

[3] Rubenstein JH, Shaheen NJ (2015). Epidemiology, Diagnosis, and Management of Esophageal Adenocarcinoma. Gastroenterology.

[4] Shapiro J, van Lanschot JJB, Hulshof M, van Hagen P, van Berge Henegouwen MI, Wijnhoven BPL (2015). Neoadjuvant chemoradiotherapy plus surgery versus surgery alone for oesophageal or junctional cancer (CROSS): long-term results of a randomised controlled trial. Lancet Oncol.

[5] Eyck BM, van Lanschot JJB, Hulshof M, van der Wilk BJ, Shapiro J, van Hagen P (2021). Ten-Year Outcome of Neoadjuvant Chemoradiotherapy Plus Surgery for Esophageal Cancer: The Randomized Controlled CROSS Trial. J Clin Oncol.

[6] Lester SC, Lin SH, Chuong M, Bhooshan N, Liao Z, Arnett AL (2017). A Multi-institutional Analysis of Trimodality Therapy for Esophageal Cancer in Elderly Patients. Int J Radiat Oncol Biol Phys.

[7] Guttmann DM, Mitra N, Metz JM, Plastaras J, Feng W, Swisher-McClure S (2018). Neoadjuvant chemoradiation is associated with improved overall survival in older patients with esophageal cancer. J Geriatr Oncol.

[8] de Geus SWL, Hirji S, Hachey KJ, Sachs TE, Suzuki K, Ng SC (2020). Lymphadenectomy and Survival After Neoadjuvant Chemoradiation for Esophageal Adenocarcinoma: Is More Better?. J Gastrointest Surg.

[9] Shridhar R, Hoffe SE, Almhanna K, Weber JM, Chuong MD, Karl RC (2013). Lymph node harvest in esophageal cancer after neoadjuvant chemoradiotherapy. Ann Surg Oncol.

[10] Camp RL (2004). X-tile: a new bio-informatics tool for biomarker assessment and outcome-based cut-point optimization. Clin Cancer Res.

[11] Marrie RA, Dawson NV, Garland A (2009). Quantile regression and restricted cubic splines are useful for exploring relationships between continuous variables. J Clin Epidemiol.

[12] Peyre CG, Hagen JA, DeMeester SR, Altorki NK, Ancona E, Griffin SM (2008). The number of lymph nodes removed predicts survival in esophageal cancer: an international study on the impact of extent of surgical resection. Ann Surg.

[13] Altorki NK, Zhou XK, Stiles B, Port JL, Paul S, Lee PC (2008). Total number of resected lymph nodes predicts survival in esophageal cancer. Ann Surg.

[14] Visser E, van Rossum PSN, Ruurda JP, van Hillegersberg R (2017). Impact of Lymph Node Yield on Overall Survival in Patients Treated With Neoadjuvant Chemoradiotherapy Followed by Esophagectomy for Cancer: A Population-based Cohort Study in the Netherlands. Ann Surg.

[15] Izbicki JR, Hosch SB, Pichlmeier U, Rehders A, Busch C, Niendorf A (1997). Prognostic value of immunohistochemically identifiable tumor cells in lymph nodes of patients with completely resected esophageal cancer. N Engl J Med.

[16] Greenstein AJ, Litle VR, Swanson SJ, Divino CM, Packer S, Wisnivesky JP (2008). Effect of the number of lymph nodes sampled on postoperative survival of lymph node-negative esophageal cancer. Cancer.

[17] Yang HX, Xu Y, Fu JH, Wang JY, Lin P, Rong TH (2010). An evaluation of the number of lymph nodes examined and survival for node-negative esophageal carcinoma: data from China. Ann Surg Oncol.

[18] Rizk NP, Ishwaran H, Rice TW, Chen LQ, Schipper PH, Kesler KA (2010). Optimum lymphadenectomy for esophageal cancer. Ann Surg.

[19] Koen Talsma A, Shapiro J, Looman CW, van Hagen P, Steyerberg EW, van der Gaast A (2014). Lymph node retrieval during esophagectomy with and without neoadjuvant chemoradiotherapy: prognostic and therapeutic impact on survival. Ann Surg.

[20] Al-Batran SE, Homann N, Pauligk C, Goetze TO, Meiler J, Kasper S (2019). Perioperative chemotherapy with fluorouracil plus leucovorin, oxaliplatin, and docetaxel versus fluorouracil or capecitabine plus cisplatin and epirubicin for locally advanced, resectable gastric or gastro-oesophageal junction adenocarcinoma (FLOT4): a randomised, phase 2/3 trial. Lancet.

[21] Zhang G, Guo X, Yuan L, Gao Z, Li J, Li X (2021). Examined lymph node count is not associated with prognosis in elderly patients with pN0 thoracic esophageal cancer. Medicine (Baltimore).

[22] Kelly RJ, Ajani JA, Kuzdzal J, Zander T, Van Cutsem E, Piessen G (2021). Adjuvant Nivolumab in Resected Esophageal or Gastroesophageal Junction Cancer. N Engl J Med.

[23] Quinn KM, Fox A, Harland KL, Russ BE, Li J, Nguyen THO (2018). Age-Related Decline in Primary CD8(+) T Cell Responses Is Associated with the Development of Senescence in Virtual Memory CD8(+) T Cells. Cell Rep.

[24] Kingma BF, Ruurda JP, van Hillegersberg R (2022). An Editorial on Lymphadenectomy in Esophagectomy for Cancer. Ann Surg Oncol.

